# Dynamic X-ray radiography reveals particle size and shape orientation fields during granular flow

**DOI:** 10.1038/s41598-017-08573-y

**Published:** 2017-08-15

**Authors:** François Guillard, Benjy Marks, Itai Einav

**Affiliations:** 1 School of Civil Engineering, The University of Sydney, Sydney, 2006 Australia; 2 Department of Civil, Environmental & Geomatic Engineering, Faculty of Engineering Science, University College London, London, WC1E 6BT UK

## Abstract

When granular materials flow, the constituent particles segregate by size and align by shape. The impacts of these changes in fabric on the flow itself are not well understood, and thus novel non-invasive means are needed to observe the interior of the material. Here, we propose a new experimental technique using dynamic X-ray radiography to make such measurements possible. The technique is based on Fourier transformation to extract spatiotemporal fields of internal particle size and shape orientation distributions during flow, in addition to complementary measurements of velocity fields through image correlation. We show X-ray radiography captures the bulk flow properties, in contrast to optical methods which typically measure flow within boundary layers, as these are adjacent to any walls. Our results reveal the rich dynamic alignment of particles with respect to streamlines in the bulk during silo discharge, the understanding of which is critical to preventing destructive instabilities and undesirable clogging. The ideas developed in this paper are directly applicable to many other open questions in granular and soft matter systems, such as the evolution of size and shape distributions in foams and biological materials.

## Introduction

After a building sinks into sand, one knows that the sand has moved, but not exactly where or how. Here, we introduce a new technique to reveal internal processes within such scenarios, common to many other flows within granular media. Granular flows encompass a wide variety of natural processes, from snow avalanches, landslides, debris and pyroclastic flows^1–4^. Industrially, they are involved in the mixing of minerals in rotating drums and the discharge of particles through hoppers and silos^5–8^. These are critical in the energy, food and pharmaceutical industries, especially where the flows present transient instabilities^9–11^ that exert dynamic forces on the supporting structure^12^. Triggered by observations from nature and industry, research in granular flows has yielded countless examples of surprising phenomena, such as the Brazil nut effect^13^, pattern formation^14^, and the jamming transition^15–17^. These effects, however, have been difficult to study experimentally due to the recurring issue of granular materials being naturally opaque. This fact has motivated the development of a number of non-invasive experimental techniques to track their internal kinematics.

Previous experimental techniques for tracking internal deformations in granular materials include X-ray computed tomography (X-ray CT)^18–23^, Positron Emission Particle Tracking (PEPT)^24–26^, Magnetic Resonance Imaging (MRI)^27–29^, ultrasonic imaging^30^, and Refractive Index-Matched Scanning (RIMS)^31–35^. Each of these techniques has associated drawbacks such as safety, cost, spatial resolution, temporal resolution, or invasiveness. Among those, X-ray CT is probably the most frequently used technique. This involves complete radiographic scanning around a sample, followed by an incremental loading step, creating delay periods. X-ray CT is therefore limited to the realm of quasi-static deformations, even though in general granular media exhibit rate-dependency. Consequently, the potential applications of X-ray CT for problems involving continuous flows are rather limited. On the other hand, RIMS has been applied to problems involving dynamic conditions, but this technique requires the use of a viscous interstitial fluid with refractive index matched to the particles under investigation, which significantly affects the nature of the granular flow^36–38^.

Faced with these limitations it is more customary to study only part of the velocity field by acquiring images solely through transparent walls or along free surfaces. Those images can then be studied using common image analysis tools applied to granular media, including Particle Image Velocimetry (PIV)^3, 35, 39, 40^ (also known as Digital Image Correlation), and Fourier related transforms^3^. However, the motion of granular media near walls tends to vary substantially from that in regions away from walls due to the formation of boundary layers^41, 42^. Therefore, it is difficult to infer the internal granular motion from measurements of the exterior of the system.

One way to minimise the effects of boundary layers along walls on the measured kinematics is the use of X-ray radiography, the building block of X-ray CT. For example, X-ray radiography has long been used to inspect density variations in a variety of geoscientific problems, including inferences about the existence of discontinuities in sandstones and clays in stationary samples^43, 44^ and measuring density in granular materials^45–47^. In addition to stationary samples, X-ray radiography was used to follow lead shot tracer particles in a soil deformed under plane strain conditions^48^. In granular media X-ray radiography was used to discover density changes within shear bands and shocks during bin and hopper flows^49–51^ and near retaining walls^52^. However, the application of this method to determine the evolution of fabric fields (such as particle size and shape orientation) in time and space has not been explored. In addition, while PIV techniques have been applied to X-ray radiography for velocity measurements in fluids using tracers^53–56^ or density fluctuations^57^, similar techniques have seldom been used for granular flows^58^.

**Figure 1:**
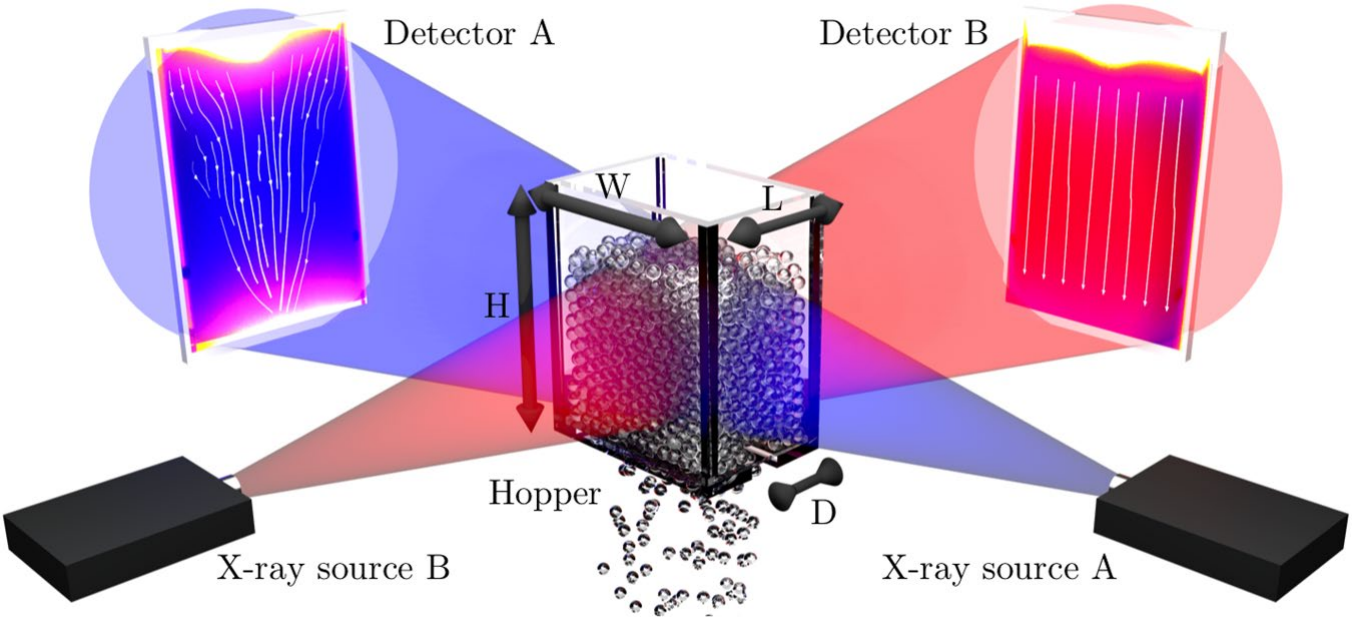
Schematic representation of the experimental setup with the two X-ray arrangements investigated here (A, shown in blue and B, shown in red). Between the source and the detector a flat-bottomed rectangular silo is placed, of size *L* × *W* × *H*, with an outlet at the base of size *D* × *W*.

The purpose of this paper is to propose a new technology for X-ray radiography of flowing granular media. The key idea is to integrate previous image analysis methods with X-ray radiography. This results in an outstanding ability to measure the comprehensive evolution of internal fabric and kinematics within flowing granular media that are translation invariant along the X-ray path, with unprecedented resolution and demonstrable accuracy. Specifically, the new technology minimises the effect of boundary layers along walls as it measures directly velocity and fabric fields within the bulk that are substantially different than those along walls, within boundary layers^41, 42^. As a canonical example we analyse the flow kinematics and fabric during silo-bin discharge with a rectangular cross section and elongated channel opening, as shown in Fig. 1. Experiments are conducted with both spherical grains (glass beads) and shape-anisotropic grains (red lentils and jasmine rice). Remarkably, the shape anisotropy will be shown to have a significant effect on the flowability of the system by promoting the ordering of elongated grains as they approach the bin’s orifice. The average orientation of the shaped particles is found to be subparallel to the streamlines and the angle between the measured flow-lines and the mean particle orientation is in agreement with recently reported measurements using X-ray tomography in discontinuous flows^59, 60^.

## Methods

In order to reveal how fabric (particle size and shape orientation) changes during granular flows, we develop in the following a technique based on Fourier transformation. To evaluate how the measured fabric relates to the bulk flow field, it is also useful to develop a PIV method applicable to dynamic X-ray radiography. The advantages of the new fabric measurement technique and the complementary velocimetry method are illustrated by monitoring the discharge of a rectangular silo, but these ideas can be adopted generally to shed light on other flow problems in granular and soft matter physics.

### Experimental configuration

The rectangular silo is described schematically in Fig. 1, and is made out of polycarbonate material to minimise X-ray absorption. The silo’s dimensions are *H* = 300 mm, *W* = 150 mm and *L* = 130 mm, with a channel opening at the bottom of width *D* = 10 mm, 12 mm or 15 mm. The experimental campaign involves testing with either glass beads, rice or red lentils. Their properties are listed in Table 1.

**Table 1:** Properties of the grains used in the experimental campaign, as measured by micrometer (*), static optical image analysis (^†^) and X-ray density wavelength, and described in the Methods section.

Material	Bulk density (kg/m^3^)	Minor axis (mm)	Major axis (mm)	Density wavelength (mm)	Aspect ratio
Jasmine rice^*^	822	1.5	6.7	2.3	4.5
Red lentils^*^	721	1.6	4.5	4.6	2.8
3 mm glass beads^†^	1,462	2.9	3.1	2.8	1.1
1 mm glass beads^†^	1,462	1.1	1.2	1.2	1.1

At the beginning of an experiment, the silo is completely filled with particles, such that the initial height is above the detector’s field of view. Discharge is initiated by releasing a trapdoor mechanism. After a brief transient period, the material discharges at a constant rate, as measured by a scale placed under the silo, as expected from the Beverloo law^61^. Temporal averages of the measured values of the fabric and velocity fields are performed during this phase of constant discharge rate. After some time, the free surface enters the field of view of the detector, and subsequently the silo fully empties.

To be able to image the flow of grains inside the apparatus, X-ray radiography is used. A Spellman XRV Generator is used to emit X-ray radiation at a maximum energy of 120 keV and intensity 3 mA for the rice and lentils, and at a maximum energy of 150 keV and intensity 5 mA for the glass beads. The radiation passes through the granular medium and the transmitted component is recorded on a PaxScan 2520DX detector at a resolution of 960 px × 768 px and at 30 frames per second.

The measured intensity *I* on a given pixel at location **x** of the detector is given as^62^

1 $$I({\bf{x}})={I}_{0}\exp ({\int }_{l}-(\mu /\rho ){\rho }_{b}(l)dl),$$

where *I*_0_ is the intensity of the source, *l* the ray path inside the medium, *μ*/*ρ* the mass attenuation coefficient (with *μ* the attenuation coefficient and *ρ* the material density) and *ρ*_*b*_ the bulk density at every location in the material. For material with homogeneous chemical composition, the radiographs therefore provide a local measurement of the bulk density of the medium averaged over the path of the ray. Flow properties are vastly modified in regions near walls known as boundary layers, for example through velocities and particle alignments substantially different than within the bulk. Therefore, in choosing geometries thicker than the typical boundary layer length^42^, X-ray radiography provides a direct measurement of the bulk properties, which are generally inaccessible using optical methods.

### X-ray velocimetry method

Since granular material are inhomogeneous in nature, the radiographs contain density fluctuations, which can be used to perform digital image correlation^63^ to measure the local displacement of a patch of material. This method assumes that the velocity of the medium is perpendicular to the rays, and does not change along the ray. These assumptions are reasonable for configuration A (see Fig. 1), since our silo is quasi-2D and produces a fairly homogeneous flow away from walls (assuming that the X-ray source produces a parallel beam). For configuration B, however, both assumptions are invalid near the opening: the velocity has non-zero components toward the centre, and the velocity is not constant since static zones exist in the corners while fast moving zones are in the middle. Indeed, the PIV fundamentally measures the modal velocity (i.e. the most probable velocity) along the beam^55^. In the following we will approximate the spatially averaged projected velocity on the plane perpendicular to the beam as the modal velocity, an assumption that will be validated in the section ‘Flow pattern’. The velocities are therefore taken as:

2 $${v}_{{\rm{m}}}\approx \frac{1}{l}{\int }_{l}({\bf{v}}-{\bf{v}}\cdot {{\bf{e}}}_{l})dl,$$

with *v*_m_ the velocity measurement from image correlation, **v** the true three-dimensional velocity field, and **e**_*l*_ the direction of the X-ray beam. This assumption has been shown to work for the case of Poiseuille flow^55^, and we will later show here that in this case, since the mean and modal velocities are close, that unless stated otherwise this assumption is in general valid.

### Fabric determination technique

**Figure 2:**
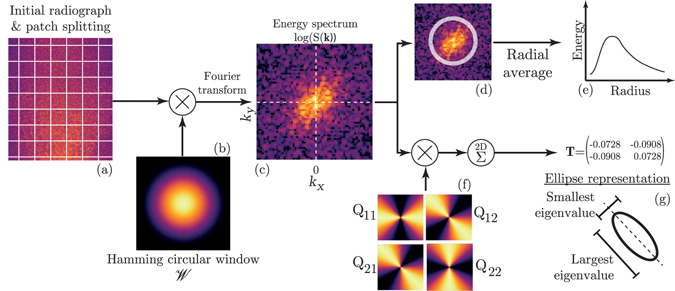
Determination method of fabric properties (particle size and orientation). From left to right: (**a**) patches of 64 × 64 pixels are extracted from a radiograph, (**b**) multiplied by a circular Hamming window, and (**c**) processed by a two-dimensional Fourier transform, leading to the power spectrum of the patch, from which two different processes are applied. Top line: (**d**) the spectrum is summed on circular shells, leading to (**e**) the energy distribution function of the radius. Bottom line: (**f**) the spectrum is multiplied by the orientation matrix **Q**(**K**) (cf. equation (4)), and summed over all wavevectors **k**, leading to the symmetric matrix **T** (cf. equation (5)), that (**g**) can also be represented by an ellipse based on its eigenvalues (ellipse shape) and eigenvectors (ellipse orientation).

In addition to the velocity, it is also possible to measure material fabric (particle size and particle shape orientation) directly from the density measurements. The size and orientation properties of the grains affect the spatial wavelength and direction of the density fluctuations on the radiograph, respectively. These properties can be conveniently recovered using a two dimensional Fourier transform. Our proposed technique employs the dynamic X-ray radiography during granular flow as sketched in Fig. 2. Each radiograph is divided into square patches of width *w* = 64 pixels, with 50% overlap between the patches. Each patch is then normalized by its average intensity and scaled by a radial hamming window ${\mathscr{W}}(r)=\,\cos (2\pi r/w)/2$ where *r* is the distance of the considered pixel from the centre of the patch. A two-dimensional Fourier transformation is then applied to each patch, as

3 $$S({\bf{k}})={|\iint I({\bf{x}})\times {\mathscr{W}}(r({\bf{x}}))\exp (2i\pi {\bf{k}}\cdot {\bf{x}})d{\bf{x}}|}^{2},$$

giving the power spectrum *S* as a function of the wavevector **k**, with *I*(**x**) the intensity of the pixel at location **x** in the patch.

From the two-dimensional power spectrum, we extract the typical size and the principal alignment direction of the particles. These fabric measurements are independent of the frame rate, and are mainly limited by the number of particles in the patch size. Larger patch size leads to greater accuracy in the orientation and size measurements, but may be unsuitable if the spatial gradients of the fabric are large.

The typical size of the particles is measured through the characteristic wavelength of the density fluctuations on the radiographs, using a orthoradial summation of the power spectrum (eg. refs 64 and 65).

The principal orientation of the particles is obtained from the power spectrum by averaging the weighted nematic order tensor. Assuming all the density fluctuations to be aligned in a patch, one would find from the power spectrum that their energy is concentrated along a single line, and that the orientation of this line would be orthogonal to the density fluctuation orientation. This can be directly used to measure fabric orientation by angular averaging^66^, but would yield insufficiently accurate results as the fluctuations get weakly oriented. In addition, such an analysis would not give any information on the intensity of the orientation. Here, we therefore advance the treatment of the power spectrum by associating to each of the power spectrum components a single structure tensor **Q** ^67, 68^. Then, by summing the weighted structure tensor, we obtain the nematic order tensor^60^. Because of the spatially discrete sampling, each component of the power spectrum at a wavevector ${\bf{k}}=({k}_{x},{k}_{y})$ effectively integrates the energy of density fluctuations in a range $[{k}_{x}\pm {\rm{\Delta }}{k}_{x}\mathrm{/2},{k}_{y}\pm {\rm{\Delta }}{k}_{y}\mathrm{/2}]$ with ${\rm{\Delta }}{k}_{x}={\rm{\Delta }}{k}_{y}=0.0156$ px^−1^ in our case. One can associate to each component of the power spectrum a structure tensor **Q**(**k**):

4 $${\bf{Q}}({\bf{k}})=\frac{1}{{\rm{\Delta }}{k}_{x}\,{\rm{\Delta }}{k}_{y}}{\int }_{{k}_{x}-{\rm{\Delta }}{k}_{x}\mathrm{/2}}^{{k}_{x}+{\rm{\Delta }}{k}_{x}\mathrm{/2}}{\int }_{{k}_{y}-{\rm{\Delta }}{k}_{y}\mathrm{/2}}^{{k}_{y}+{\rm{\Delta }}{k}_{y}\mathrm{/2}}\frac{{\bf{k}}\otimes {\bf{k}}}{{|{\bf{k}}|}^{2}}{\rm{d}}{\bf{k}}\mathrm{.}$$

The norm of the matrix **Q** is generally close to 1 for small wavelengths (corresponding to large wavevectors, $|{\bf{k}}|\gg {\rm{\Delta }}{k}_{x},{\rm{\Delta }}{k}_{y}$), and decreases for larger wavelengths, since the power spectrum components integrate the energy in a space of directions that is more scattered (*k*_*x*_ ~ Δ*k*_*x*_ and *k*_*y*_ ~ Δ*k*_*y*_). This orientation matrix is then weighted by the power spectrum components leading to the two-dimensional nematic order tensor for the considered patch

5 $${\bf{T}}=\sqrt{2}\times (\frac{\sum _{{\bf{k}}}S({\bf{k}}){\bf{Q}}({\bf{k}})}{\sum _{k}S({\bf{k}})}-\frac{1}{2}{\bf{I}}),$$

where **I** is the identity matrix. The matrix **T** defined in this way is traceless, and we define the ordering parameter as $\Vert {\bf{T}}\Vert =\sqrt{{T}_{ij}{T}_{ij}}$, with $\Vert {\bf{T}}\Vert =0$ for an isotropic power spectrum (no preferred orientation) and ||**T**|| = 1 for a power spectrum where all the energy is in only one direction. To reduce noise from the power spectrum, we also average **T** over time to get $\overline{{\bf{T}}}$. The time window is chosen to be large enough to remove fluctuations in $\overline{{\bf{T}}}$ but small enough so that **T** is quasi-steady during this time. This is easily achievable for the flows studied in this paper. The principal orientation of the power spectrum is directly given by the eigenvector of **T** or $\overline{{\bf{T}}}$ associated with its largest eigenvalue, as shown in Fig. 2g.

## Results

### Flow field measurement

**Figure 3:**
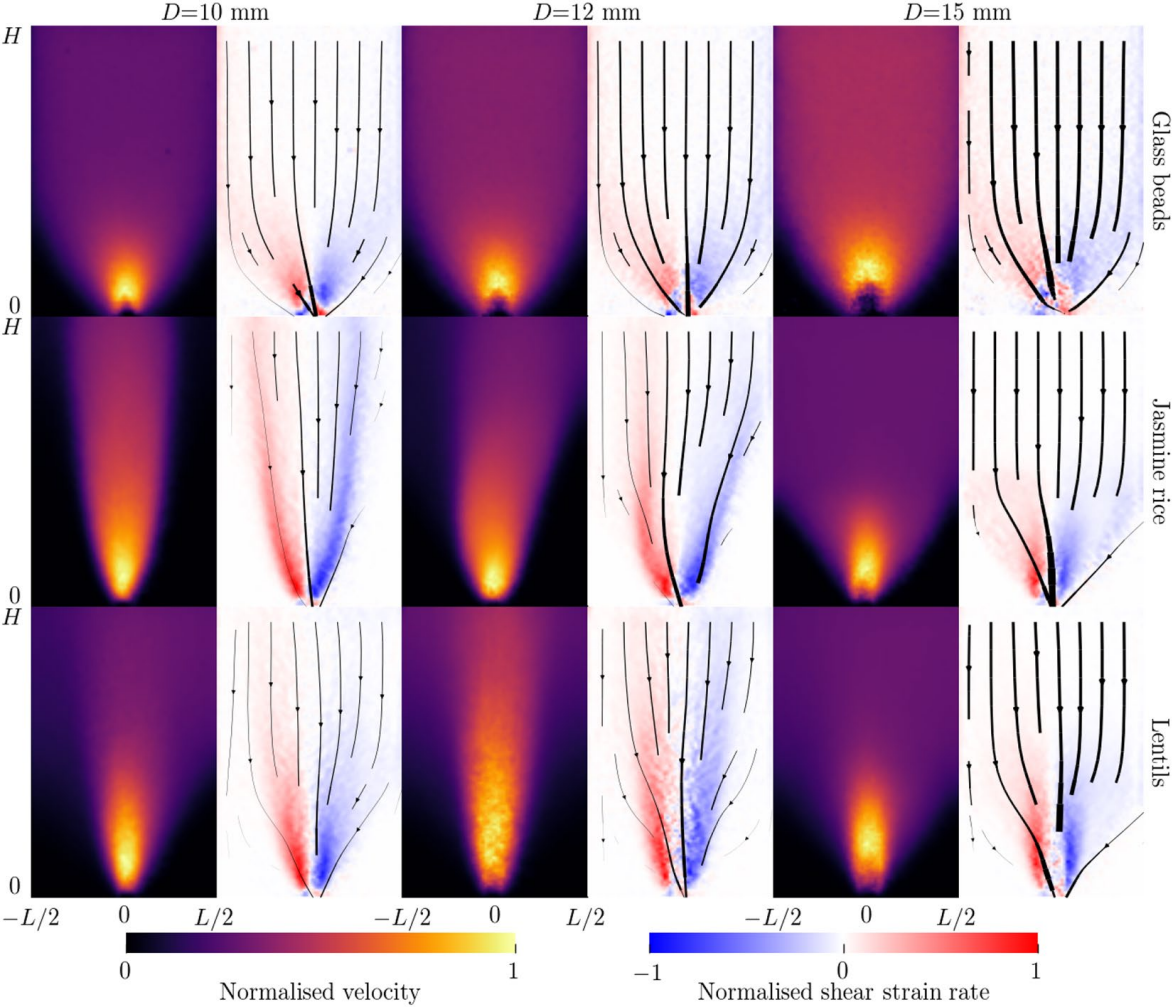
Velocity magnitude and shear strain rate during developed flow, as measured from detector A. Black lines indicate streamlines of the velocity field, with thickness representing magnitude. *Left to right: D* = 10, 12 and 15 mm. *Top:* Glass beads. *Middle:* Jasmine rice. *Bottom:* Red lentils. *Left to right*, *top to bottom:* Normalisation velocity is 28.4, 37.4 and 49.6 mm/s; 18.4, 37.5 and 56.3 mm/s; 17.6, 22.6 and 65.2 mm/s.

Figure 3 shows the velocity magnitude and shear strain rate as measured using PIV for radiographs along projection A, for the three materials and three outlet openings, after averaging over time in the steady-state regime. For this silo geometry, we observe both funnel and mass flow regimes, depending on the material and outlet size, with funnelling tending to be stronger for smaller openings and non-spherical grains. The glass beads flow uniformly (i.e. in a relatively constant manner over time), whereas the flow for the elongated particles exhibits some transient instability. In these cases, where funnel flow is present, the velocity field is not constant, but oscillates over time. Please see the Supplementary Material for videos of this instability.

**Figure 4:**
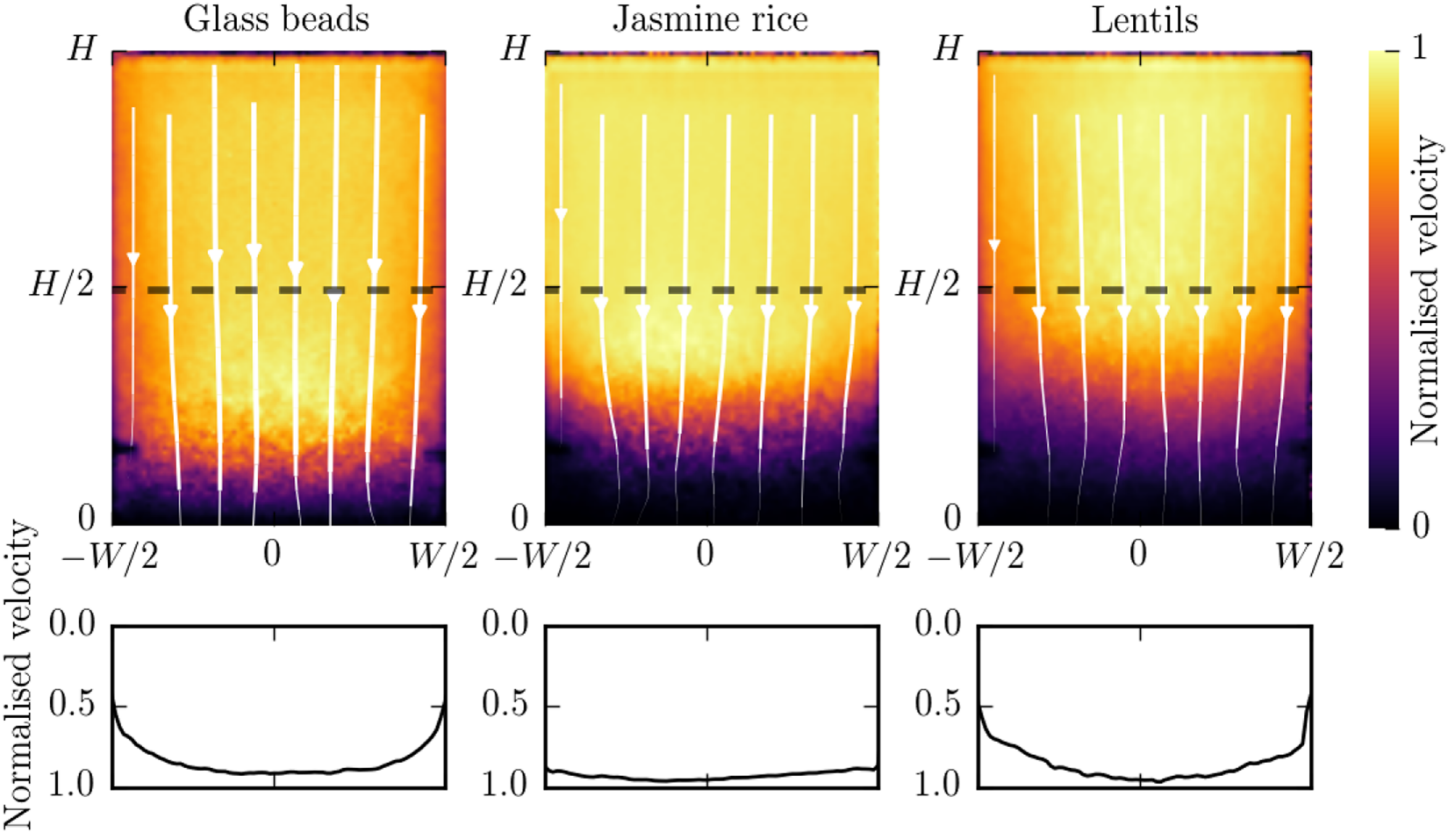
Velocity field during developed flow measured from detector B for the largest outlet, *D* = 15 mm. *Top:* Normalised velocity magnitude and streamlines (in white). *Bottom*: Normalised vertical velocity along the dashed lines. *Left to Right:* Glass beads, jasmine rice and red lentils. Normalisation velocities are 24.6, 26.4 and 27.1 mm/s, respectively.

Figure 4 indicates the velocity magnitude measured using projection B. The lower magnitude of the velocity at the bottom can be explained by the projection effect described in equation (2). Near the opening, the flow converges toward the channel and therefore has a non-negligible component along the X-ray direction in projection A. The velocity magnitude measured at the bottom is therefore much smaller than one would expect. Interestingly, aside from this bottom part, the velocity magnitude is independent of the vertical location, even where there is a funnelling effect that changes the width of the flowing zone. Also, note that the velocity is smaller near the walls due to the development of boundary layers^42^, which highlights the advantage of measuring velocities using dynamic X-ray radiography rather than conventional PIV of photographic images taken along flow boundaries.

**Figure 5:**
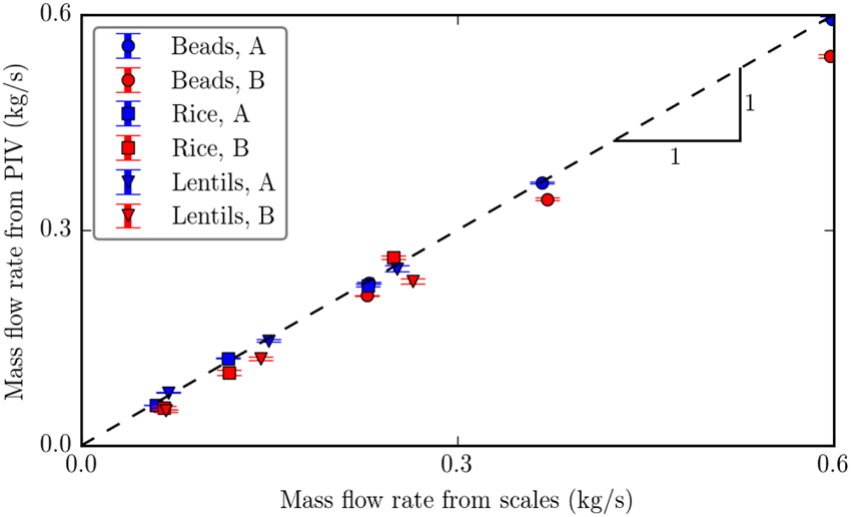
Comparison of the mass flow rate measured by PIV and by mass discharge measurements, for three outlet sizes and three materials, as measured from both A and B projections. The dashed line represents a slope of unity, for which the two measurements are in agreement.

To quantitatively assess that the velocity measured using image correlation is indeed the bulk velocity, we compare the flow rate obtained from integration of the velocity along the width of the silo with the discharge flow rate measured by the scale below the silo. In a steady discharge state (where the flow rate is constant in time) these two measurements should be equal. Figure 5 compares the flow rate measured from DIC at individual heights between the middle of the recorded image (122 mm from the base) and 200 mm from the base for the two projections A and B in the silo, for the three materials with the discharge rate measured on the scale. Error bars represent the standard deviation of these measurements for each tested flow geometry.

The good agreement between the data in Fig. 5 indicates that the PIV method is valid for measuring velocities in this two-dimensional configuration. In particular, this confirms that the assumption leading to equation (2) is reasonable, at least for the present experiments.

### Fabric measurement

**Figure 6:**
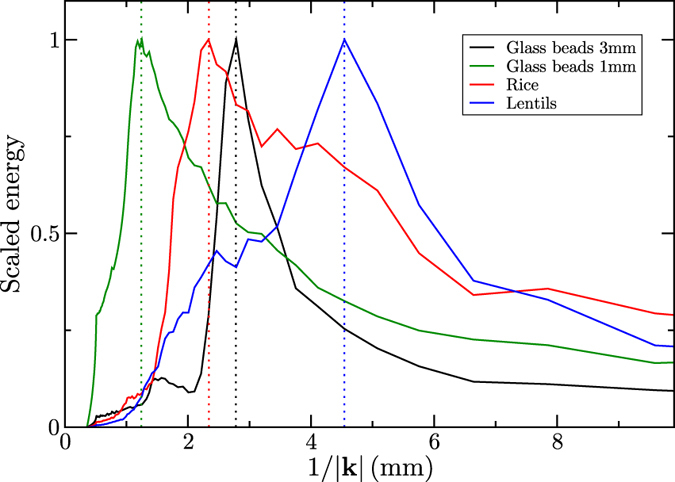
Energy scaled by peak energy function of the fluctuation wavelength for the three materials, measured by radial averaging of the power spectrum obtained by Fourier transform. Dotted lines indicate the peak locations.

Following the method described in the section ‘Methods’, the typical size of the density fluctuations of the radiographs is measured using a Fourier transform method. Figure 6 shows the radially summed energy spectrum as a function of the wavelength, for the glass beads, rice and lentils. Each of the spectra has a peak at a characteristic wavelength, indicated with a dotted line, corresponding to the typical particle size, and reported in Table 1 under “density wavelength”. The size of the lentils and rice have also been measured independently by micrometer from 20 randomly chosen particles of each type. The glass bead sizes were measured using static image analysis in a Malvern Morphologi G3. The size measured from the spectrum is very close to the measured diameter of the glass beads. Lentils and rice, however, have two characteristic dimensions, which explains the broader distributions in Fig. 6. Taking the shape of the particles to be ellipsoidal, the rice has two equal minor axes and one major axis, and the lentils the opposite. Because the particles are not perfectly aligned, we find a range of lengths in the Fourier transform, which are representative of their cross section perpendicular to the X-ray beam. This explains why the characteristic wavelength for the density fluctuations is close to the small dimension of the rice, and close to the large dimension of the lentils (Table 1). For particles with a small aspect ratio, measuring the typical wavelength of the density fluctuations of the radiographs using a Fourier transform gives a good indication of the size of the particles involved in the flow, which can be of interest for flows involving polydisperse particles.

**Figure 7:**
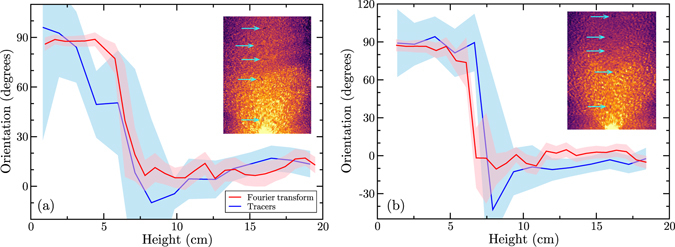
Comparison between orientation measured from tracers or by 2D Fourier transform of the density field for rice (**a**) or lentils (**b**). Shaded area are the 95% confidence interval on the estimated mean angle^69^. Insets: examples of radiographs of the silo flows with tracer particles being indicated by the blue arrows.

**Figure 8:**
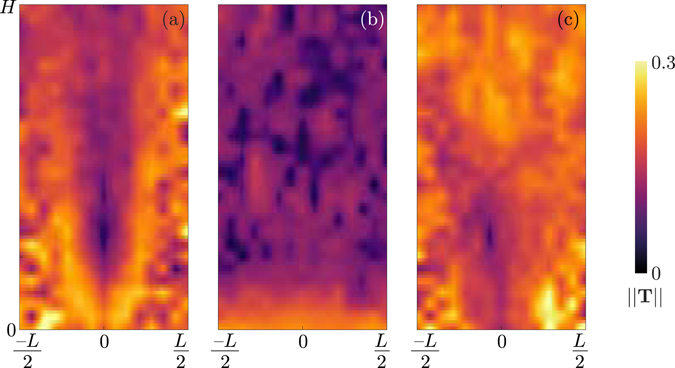
Order parameter field for (**a**) jasmine rice, (**b**) glass beads, (**c**) red lentils, for the case of *D* = 10 mm opening.

As described in the section ‘Methods’, it is possible to obtain the characteristic orientation of the grains from the density fluctuations by using a Fourier transform. Firstly, we assess the validity of this method by comparing its result to direct measurement of the orientation of steel tracer particles seeded within the flow. During the filling of the silo, tracer particles are placed approximately every 2 cm vertically above one another, in the centre of the silo. These tracers are of similar size to the grains in the medium, but their metallic composition increases their X-ray attenuation, making them easily visible in comparison to the surrounding particles. The tracers can therefore be tracked individually. Assuming that their orientation at any given time is similar to the orientation of the surrounding grains, the tracers allow for the tracking of the average particle orientation. Figure 7 shows a comparison between the tracer orientation and the orientation measured by Fourier transform along the central line (eigenvector associated to the largest eigenvalue of the matrix **T**). The good agreement between the two measurement methods indicates that the Fourier transform can be used to estimate local orientation of the granular material at any position in space and time, much more efficiently than by using tracer particles. Figure 8 shows the order parameter ||**T**|| for the three materials, with the smallest opening, *D* = 10 mm. The glass beads do not show any ordering during the flow, which is expected since they are isotropic (Fig. 8b). Note that the apparent increase of ordering at the bottom of the silo is an artefact of the X-ray measurement: X-ray scattering is particularly visible at the bottom of the silo, creating a gradient of intensity that is picked up by the processing as corresponding to a preferred orientation. This effect is particularly visible in the glass beads because of their lack of orientation and their higher X-ray absorption coefficient. Figure 8a and c show that the order parameter is much higher for the rice and lentils, in particular in the zone of high shear (Fig. 8a) where the funnel geometry of the flow is clearly visible (compare Fig. 8a to middle left panel of Fig. 3).

**Figure 9:**
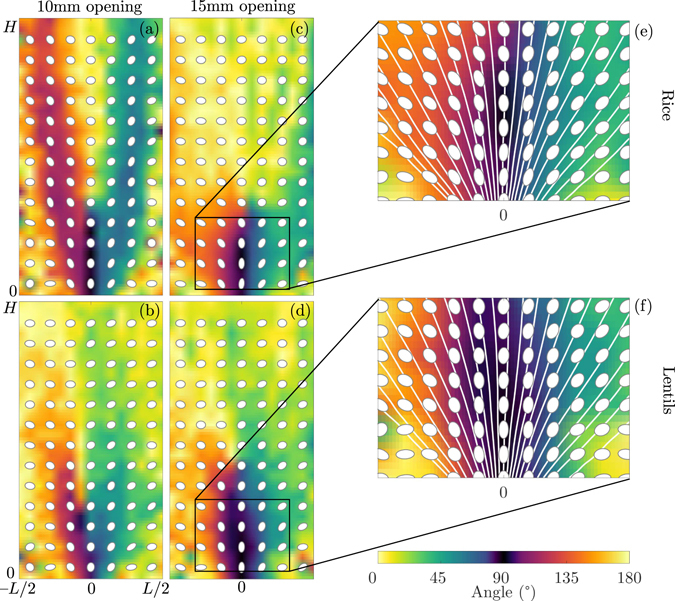
Principal particle orientation for rice (**a**,**c**,**e**) and lentils (**b**,**d**,**f**) measured from Fourier transforms. Colormaps represent the principal angle and ellipses represent the tensor **T** at their location. (**a**–**d**) Full field view of the silo, (**a**,**b**) 10 mm opening, (**c**,**d**) 15 mm opening. (**e**,**f**) Zoom near the outlet of the 15 mm opening, as indicated by the black rectangles in (**c**,**d**), with streamlines computed from Fig. 3.

Figure 9 shows maps of the particle-shape orientation for the rice and lentils, at small and large openings. The overlaid ellipses are representations of the matrix **T**, as described in Fig. 2g. All experiment are approximately symmetric along the centre line, as expected and suggested by the velocity fields Fig. 3. The funnelling effect and the alignment of the particles in the sheared zones is also recovered for the small opening, whereas for the large opening the flow is essentially a bulk flow above a height of ~10 cm where the grains are typically lying horizontally because of the filling procedure. When the grains reach zones of higher shear near the opening (height <10 cm), they begin to align with the flow, but do not perfectly align parallel to the streamlines, as discussed previously by ref. 59. This effect is shown in Fig. 9e and f which are zoomed views of the behaviour near the opening and depict the streamlines computed from the velocity field measured by PIV.

## Discussion

Using a newly developed technique, this paper demonstrates how dynamic X-ray radiography can be used to quantitatively measure velocity and fabric fields during flows of granular media. The use of X-ray radiography presents several significant advantages. Firstly, the contribution of each particle to the bulk motion is weighted equally due to the nature of X-ray attenuation. Therefore, for two dimensional flows, the influence of wall effects on the measurements can be decreased simply by increasing the dimension of the experiment along the X-ray path, since the bulk fields (of velocity and fabric) contribute to their averages more than the corresponding fields within the boundary layers. Secondly, radiography allows for the tracking of rapid flows, as detector panel technology now allows for recording at up to approximately 100 Hz. Thirdly, by measuring the modal velocity across a device, wall effects can be significantly minimised. The success of the proposed velocimetry method and the fabric determination technique are validated by the fact that both give results that are in good agreement with the conventional techniques of mass flow-rate measurement and tracer tracking, respectively.

There are many interesting problems which may be studied with such methods. For example, in biological systems the deformation, orientation and density of red blood cells may be tracked during flow, especially in complex geometries such as around aneurysms^70^. In a geological context, the spatial inhomogeneity of grains during debris flows, landslides and avalanches due to segregation and grain crushing may also be quantified^71^. Similar issues are present in industrial operations, such as vibrated beds of powders and pharmaceutical tabletting, where the coupled ordering and flow of powders present significant design challenges.

Moreover, numerous industrial processes involve the addition of particles or fibres to improve the mechanical properties of products^72^. The spatial distribution and orientation of these have a controlling influence on the performance of the material. Being able to dynamically measure the fibre orientation in the matrix during flow can provide key insights into the mechanical performance of the composite or suspension towards improving the fabrication process, without relying on optically matched suspensions^73^ or tracer additives^74^. Finally, it is possible to extend these methods to obtain the full three-dimensional velocity or fabric fields, using multiple X-ray projection and reconstruction techniques such as computed tomography^55^. These methods give new information and insights into the physics of granular material that were previously inaccessible.

## Electronic supplementary material

Supplementary video, glass beads discharge D=10mm

Supplementary video, glass beads discharge D=15mm

Supplementary video, lentils discharge D=10mm

Supplementary video, lentils discharge D=15mm

Supplementary video, rice discharge D=10mm

Supplementary video, rice discharge D=15mm
